# Changes in the Bispectral Index in Response to Experimental Noxious Stimuli in Adults under General Anesthesia

**DOI:** 10.1155/2013/583920

**Published:** 2013-08-01

**Authors:** Robin Marie Coleman, Yannick Tousignant-Laflamme, Céline Gélinas, Manon Choinière, Maya Atallah, Elizabeth Parenteau-Goudreault, Patricia Bourgault

**Affiliations:** ^1^School of Nursing, Faculty of Medicine and Health Sciences, University of Sherbrooke, Sherbrooke, QC, Canada J1K 2R1; ^2^Sherbrooke University Hospital (CHUS), Sherbrooke, QC, Canada J1H 1P8; ^3^School of Rehabilitation, Faculty of Medicine and Health Sciences, University of Sherbrooke, Sherbrooke, QC, Canada J1K 2R1; ^4^Centre de Recherche Clinique Étienne-Le Bel du Centre Hospitalier Universitaire de Sherbrooke (CHUS), Sherbrooke, QC, Canada J1H 5N4; ^5^School of Nursing, McGill University, Montreal, QC, Canada H3A 0G4; ^6^Center for Nursing Research and Lady Davis Institute, Jewish General Hospital, Montreal, QC, Canada H3T 1E2; ^7^Department of Anesthesiology, School of Medicine, University of Montreal, Montreal, QC, Canada H3C 3J7; ^8^Department of Anesthesiology, School of Medicine, Faculty of Medicine and Health Sciences, University of Sherbrooke, Sherbrooke, QC, Canada J1K 2R1

## Abstract

*Objective*. Pain assessment is a major challenge in nonverbal patients in the intensive care unit (ICU). Recent studies suggest a relationship between the Bispectral Index (BIS) and nociceptive stimuli. This study was designed to examine changes in BIS in response to experimental noxious stimuli. *Methods*. Thirty participants under general anesthesia were in this quasiexperimental, within subject, pre- and poststudy. In the operating room (OR), BIS was monitored during moderate and severe noxious stimuli, induced by a thermal probe on the participants' forearm, after induction of general anesthesia, prior to surgery. *Results*. Significant increases in BIS occurred during moderate (increase from 35.00 to 40.00, P = 0.003) and severe noxious stimuli (increase from 37.67 to 40.00, P = 0.007). ROC showed a sensitivity (Se) of 40.0% and a specificity (Sp) of 73.3% at a BIS value > 45, in distinguishing a moderate from a severe noxious stimuli. *Conclusion*. BIS increased in response to moderate and severe noxious stimuli. The Se and Sp of the BIS did not support the use of the BIS for distinction of different pain intensities in the context of deep sedation in the OR. However, the results justify further studies in more lightly sedated patients such as those in the ICU.

## 1. Introduction

Pain is commonly experienced during a stay in the intensive care unit (ICU). It is recognized that several procedures such as turning, endotracheal suctioning, and drain removal are painful, even for intubated and sedated patients [1]. However, pain assessment is a major challenge for health professionals since several barriers limit patients' verbal communication and their ability to self-report pain [1–4]. In those who can communicate, up to 80% report having suffered moderate to severe pain during their ICU stay, suggesting an urgent need to enhance pain management in the context of critical care [1, 3, 5]. Pain under treatment may lead to a number of adverse physical consequences including respiratory complications [5–7]. In addition, when acute pain is inadequately relieved, it may contribute to increase the risk of developing chronic pain [8, 9]. This can have a serious impact on the individual's level of functioning and cause great emotional distress, hindering quality of life, and long-term well-being [10]. Such findings strengthen the importance of improving pain assessment and management in the ICU.

The American Society of Pain Management Nursing (ASPMN) published clinical recommendations pertaining to pain assessment in nonverbal patients, such as those who are unconscious or sedated and mechanically ventilated in the ICU [11, 12]. When the patient's condition prevents subjective assessment, the ASPMN [12] recommends the use of a valid behavioral scale for detection of pain such as the Critical-Care Pain Observation Tool (CPOT) [13] or the Behavioral Pain Scale (BPS) [14]. However, considering that pain behaviors can be attenuated by factors, such as deep sedation and the use of neuromuscular blockade agents (NMBA), they convey a limit which needs to be addressed [13–20]. Therefore, the time has come to investigate noninvasive, innovative, and physiological measures with potential use for the detection of pain at the patient's bedside such as those relating to cortical activities and facial expressions. The bispectral index (BIS) is one of such measures and is newly studied in the field of pain.

The BIS (Aspect Medical Systems, Newton, MA, USA) is a noninvasive device reflecting the electrical activity of the cortex and of the electromyographical (EMG) activity of temporal and forehead muscles (corrugator supercilii muscle). It is a weighted sum of electroencephalographic (EEG) subparameters computed from a composite of several measures from EEG signal processing techniques. The BIS is a dimensionless number, scaled to correlate with important clinical states, varying from 0 (an isoelectric electroencephalogram or profound anesthesia) to 100 (fully awake clinical state) [21–23]. The BIS was approved by the United States Food and Drug Administration in 1996 for monitoring sedation levels in the operating room (OR). Even though BIS has primarily been studied in the context of sedation, several studies suggest a possible relationship between BIS and nociceptive procedures in demonstrating that the BIS value tends to increase during nociceptive stimuli [22, 24–26]. Furthermore, a recent pilot study in sedated and mechanically ventilated adults in the ICU compared BIS responses at rest (baseline) and during turning and endotracheal suctioning, two procedures known to be painful [18]. Significant increases in BIS were observed during both nociceptive procedures when compared to baseline measures (median BIS increased by 20–30%) [18]. While these studies show the potential utility of the BIS for detection of pain in the ICU, further research is warranted to determine if BIS increases were specific to noxious stimuli [18, 22, 24–27].

Hence, the specific objectives of this study were to (1) describe the changes in BIS in response to experimental noxious stimuli of moderate (40/100) and severe (70/100) intensities and (2) examine the sensitivity and specificity of the BIS in distinguishing noxious stimuli of different intensities (moderate and severe). An accessible population which allowed the control of several variables was sought and sedated, and mechanically ventilated adults under general anesthesia in the OR were selected.

## 2. Materials and Methods

### 2.1. Design, Setting, and Ethics

A quasiexperimental, within subject, pre- and poststudy design was used. The study was conducted at the Centre Hospitalier Universitaire de Sherbrooke, Sherbrooke, (QC, Canada). Ethics approval was obtained by the Local Ethics Review Board of the institution (Centre de Recherche Clinique Étienne-Lebel du Centre Hospitalier Universitaire de Sherbrooke). All participants gave written informed consent to participate in the study and were provided with a free parking pass. Participants were informed that they could withdraw from the study at any moment up until general anesthesia.

### 2.2. Participants

Potential participants were met by a member of the research team during the preoperative clinic visit a few days before surgery or upon admission the day of surgery to explain the study and obtain written consent. Information on medication, age, and allergies was obtained to verify eligibility criteria. Eligible patients were between 18 and 70 years old, waiting to undergo elective general or orthopedic surgery under general anesthesia and classified as ASA I or ASA II on the American Society of Anaesthesiologists Physical Status Classification System, that is, normal healthy patients or presence of mild systematic diseases such as hypertension or diabetes with no systemic effects [28]. Patients were excluded if they were taking opioids in the preoperative period, to avoid alterations in pain perception. Those who presented an allergy to fentanyl or morphine derivatives were also excluded to ensure applicability of the standardized medication protocol during anesthesia induction in the OR.

### 2.3. Noxious Procedures

The independent variables were the thermal noxious stimulus determined by each participant as being moderate (*X*_1_) and severe (*X*_2_) in intensity. Moderate noxious stimuli (*X*_1_) was defined as the thermal noxious stimuli of 40/100 in intensity, and the severe noxious stimuli (*X*_2_) was defined as the thermal noxious stimuli of 70/100 in intensity. The temperature of the thermal probe for each stimulus (*X*_1_  and  *X*_2_) was precisely determined by each subject with the help of Thermal Pain Stimulator (TPS) and a Computerized Visual Analog System (CoVAS). The sequence consisted of two noxious stimuli (*X*_1_  and  *X*_2_) of 30 seconds each, preceded by a 30 second baseline measurement (*B*_1_  and  *B*_2_) with the temperature of probe at 32°C (room temperature).

## 3. Apparatus

### 3.1. Thermal Pain Stimulator (TPS)

The TPS (TSA II) from Medoc-Advanced Medical Systems was used to induce thermal noxious stimuli and was connected to a Computerized Visual Analog Scale (CoVAS). The thermal probe is 9 cm² and consists of a peltier element, a contact plate, three thermistors, a cooling element plaque, and a Velcro attachment to secure the probe on the subjects arm. The TPS administered a heat stimulus to a participant's forearm with a rising rate of 0.3°C per second. Internal safety mechanisms safeguard the patient by preventing the probe from surpassing a temperature of 51°C during heating tests. No electrical inference has been documented between the TPS and BIS.

### 3.2. Computerized Visual Analog Scale (CoVAS)

Thermal pain intensity was measured with the CoVAS, from Medoc-Advanced Medical Systems. It was used by participants to determine independent variables (*X*_1_  and  *X*_2_). During thermal noxious stimuli, the participant moved the slide on the CoVAS according to pain intensity. The slide was adjusted to a predetermined level of 0 (no pain) to 100 (maximum imaginable pain). The results were simultaneously recorded on the computer in chart form, containing information related to the specific time of the stimulus, the temperature of the probe, and the score on the CoVAS.

## 4. Main Outcome Measurement

### 4.1. BIS

The BIS was the only outcome measure in this study. It was acquired with the Bispectral Index Monitor. Reliability and validity of the Bispectral Index Monitor have previously been established for sedation purposes [29, 30]. BIS was recorded continuously with the portable Bispectral Index Monitor (Model A-2000 software version 3.20, Aspect Medical System), used according to the manufacturer's instructions. To ensure appropriate use of the BIS, the research assistant followed an online training, available on the manufacturer's website [31].

## 5. Procedure

The procedure occurred during the preoperative period and in the OR as described in the following.

### 5.1. Preoperative Period

#### 5.1.1. Familiarization with Experimental Procedures and Pain Ratings

During this first encounter with the participants at the preoperative clinic, a familiarization period with the TPS and the CoVAS was performed. The research assistant applied the TPS on the participants' forearm, and they were informed that the temperature of the TPS would gradually increase. Participants were instructed to evaluate pain intensity by moving the slide on the CoVAS and that the procedure would stop when the maximum thermode temperature tolerated was identified by the participant (CoVAS score of 100/100).

### 5.2. Operating Room (OR)

#### 5.2.1. Preparation

The BIS sensor was applied on participant's forehead and temporal region by research assistant. The sensor was connected to the BIS Monitor via the interface cable. The thermal probe was then placed on participants' nondominant forearm with a Velcro attachment. Participants were administered a dose of 2-3 mcg/kg of fentanyl intravenously (IV), by the anesthesiologist in accordance with the practice in the institution. To achieve peak plasma concentration of fentanyl [32], a seven minute waiting period was initiated with a stopwatch. After this waiting period, the thermal noxious stimuli were administered to the participants' forearm. While receiving the stimuli, the participant evaluated their pain intensity with the CoVAS, identifying the precise temperatures of the thermal probe that induced noxious stimuli rated as moderate (*X*_1_) and severe (*X*_2_) in intensity.

#### 5.2.2. Induction of Anesthesia

**Figure 1:**
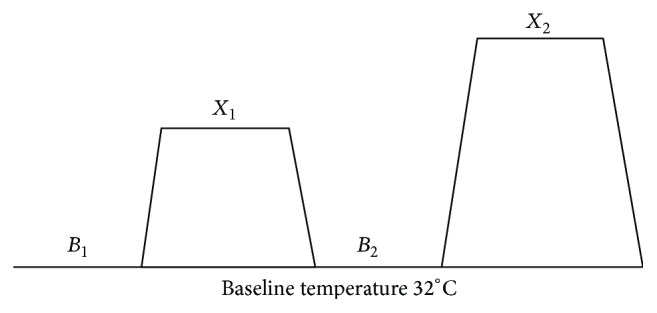
Experimental noxious stimuli (*X*_1_  and  *X*_2_). *B*_1_: baseline 1; *B*_2_: baseline 2; *X*_1_: moderate noxious stimuli (40/100); *X*_2_: severe noxious stimuli (70/100). Each observation period was 30 seconds in duration. BIS was continuously monitored, and values were documented at 10 second intervals (3 values for each 30 seconds observation period). The maximum BIS value for each 30 second observation period was used for analysis.

Following determination of independent variables (*X*_1_  and  *X*_2_), the location of thermal probe on the participants' forearm was changed. The anesthesiologist administered induction medication and performed endotracheal intubation. The medication protocol, based on local practice of anesthesiologists consisted of propofol 2-3 mg/kg, rocuronium 0.6 mg/kg [32], or succinylcholine 1-2 mg/kg with a dose of rocuronium of 0.03 mg/kg [33]. Before inducing the noxious stimuli, a stable baseline BIS value between 20 and 50 needed to be obtained. This range of values was chosen in accordance with local sedation level practice and represents levels of sedation varying from a deep hypnotic state (20–40) to a state of general anesthesia (40–60) [34]. Type and dose of medication administered by the anesthesiologist for induction of anesthesia such sedative agents, opioids, and neuromuscular blockade agents (NMBA) were noted as well as the value of the minimum alveolar concentration of anesthetic (MAC) at the start of the experimental phase. Once a stable baseline BIS value was achieved, the experimental phase and observations began (Figure 1).

### 5.3. Sample Size

Sample size was calculated using the n Query Advisor, software version 4.0. Power analysis based on a clinically important difference of 5 of the BIS value with a standard deviation of 10, indicated that 30 participants were needed to detect a difference with 80% power and 95% confidence. Sample size was confirmed by calculations based on the preliminary results of a previous study [18].

### 5.4. Data Analysis

Considering that our data was not normally distributed, nonparametric tests were used for all comparisons. Furthermore, descriptive statistics are presented as median scores and interquartile ranges (IQR), except for medication. All doses of each type of medication administered for anesthesia were calculated in relation to the participant's weight. The individual doses were then grouped together to provide a mean dose and a standard deviation.

To examine changes in BIS values during noxious stimuli, we compared the maximum BIS values (the highest BIS value during the 30 second interval) for all participants during *B*_1_ to the maximum BIS for all participants during *X*_1_ using the Wilcoxon signed-rank test (for related samples). The same analysis was performed to compare the maximum BIS for all participants during *B*_2_ observations to the maximum BIS for all participants during *X*_2_.

To examine within subject changes in BIS during noxious stimuli, we calculated the difference between the maximum BIS during *X*_1_ and the maximum BIS during *B*_1_ (Δ1). The same analyses were performed to calculate the difference between *X*_2_ and *B*_2_ (Δ2) BIS observations. Deltas 1 and 2 were compared using the Mann-Whitney *U* Test (nonparametric) for independent samples to determine if there was a significant difference in BIS changes in relation to the intensity of noxious stimuli.

To evaluate the ability of the BIS to distinguish a moderate from severe noxious stimuli, Se and Sp were performed with a Receiver Operating Characteristic Curve (ROC) analysis. The ROC, Area Under the Curve (AUC), confidence interval, and Z statistic were examined [35].

#### 5.4.1. Complementary Analyses

Several factors proven to modify BIS must be considered in studying this potential relationship. It has been shown that administration of neuromuscular blockade agents (NMBA) will diminish the expected increase of BIS values in response to noxious stimuli [36]. Furthermore, higher doses of sedative agents such as propofol are known to correlate with lower BIS (*r* = −0.559, *P* < 0.001) [37]. It has also been proven that changes in BIS significantly differ depending on the dose and type of opioid administered (i.e., Fentanyl) [38]. As well, in studies with participants under general anesthesia, it has been shown that minimum alveolar concentration of anesthetic (MAC) must be around 1.0 to 1.3 to observe an increase in BIS during painful stimuli [26, 39]. The MAC is the alveolar concentration of anesthetics necessary to prevent a motor response in 50% of patients in response to surgical pain [26, 39].

#### 5.4.2. Neuromuscular Blockade Agents (NMBA)

Two possible doses of NMBA (rocuronium) were permitted in the research protocol. Therefore, further analyses were performed in relation to the dose of NMBA received to determine if the different doses confounded results. Participants were divided into one of two groups having received either a dose of >0.03 mg/kg or ≤ of 0.03 mg/kg. To examine the presence or absence of a difference in BIS, the maximum BIS of the participants in the two groups was compared at each of the four measurement periods (*B*_1_, *X*_1_, *B*_2_, and  *X*_2_), using Mann-Whitney *U* Test (nonparametric) for independent samples.

#### 5.4.3. Sedation Level

In accordance with protocol, a range of BIS from 20 to 50 represented the stable BIS score that was sought before start of the baseline observations. Therefore, similar analyses were performed in relation to the sedation level of the participants to determine if the variation in sedation levels resulted in different changes in BIS. Participants were divided into one of two groups according to whether *B*_1_ and *B*_2_ were >40 (state of general anesthesia) or <40 (deep hypnotic state) at stable baseline BIS value for each of the two noxious stimuli. The maximum BIS of the participants in the two groups was then compared for each of the two noxious stimuli (*X*_1_  and  *X*_2_), using Mann-Whitney *U* Test (nonparametric) for independent samples.

## 6. Results

### 6.1. Sample Characteristics

A convenience, nonprobabilistic sample of 32 participants who fulfilled the eligibility criteria completed the study. However, two participants had to be excluded during the experimental procedures since they did not have a stable baseline BIS value between 20 and 50. Therefore, 30 participants were retained for data analysis, including 22 women and 8 men. The mean age of the participants was 52.93 (s.d. ± 12.50). Twenty-nine participants were scheduled to undergo a general surgery, while the others were scheduled to undergo an orthopedic surgery. Description of medication and MAC administered to participants is presented in Table 1.

#### 6.1.1. Changes in BIS in Response to Moderate and Severe Experimental Noxious Stimuli

To examine changes in BIS during noxious stimuli, we compared maximum *B*_1_ or *B*_2_ observations for all participants with their corresponding max BIS observations during each the noxious stimuli. There was a statistically significant increase of BIS during both noxious stimuli (Table 2). The median BIS increased by 12.5% during the moderate stimulation (*P* = 0.003) and by 8.3% during the severe stimulation (*P* = 0.007).

To examine within subject changes in BIS during each noxious stimuli (*X*_1_  and  *X*_2_), the difference between *X*_1_ and *B*_1_ (Δ1) and the difference between *X*_2_ and *B*_2_ (Δ2) were calculated. The median and IQR for Δ1 and Δ2 BIS observations are presented in Table 3. Further analysis showed that there was no significant difference between these two increases (*P* = 0.847) which suggest that both thermal noxious stimuli induced comparable increases in BIS value.

#### 6.1.2. Sensitivity (Se) and Specificity (Sp)

**Figure 2:**
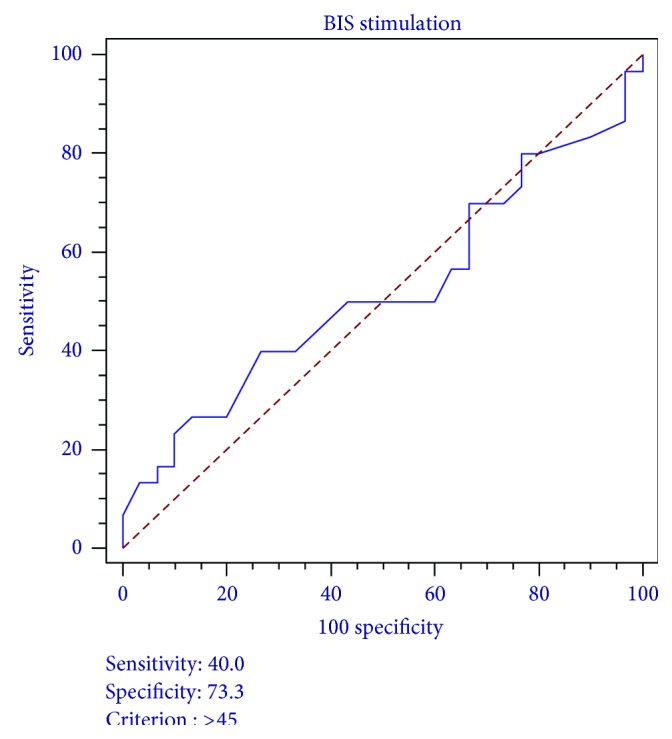
ROC curve BIS values and the distinction of moderate and severe noxious stimuli. ROC curve analysis graph with BIS > 45 (the threshold associated with the maximization of sums of sensitivity and specificity of BIS in the distinction of moderate and severe noxious stimuli).

The threshold associated with maximization of the sums of sensitivity and specificity was found to be BIS > 45 (Table 4). For this criteria, ROC analysis determined a Se of 40.0% and a Sp of 73.3% for BIS in distinguishing a moderate noxious stimulus (*X*_1_) from a severe noxious stimulus (*X*_2_). The ROC curve is shown in Figure 2. The area under the curve is 0.521 with a standard error of 0.0768, a 95% confidence interval ranging from 0.0388 to 0.6520 (*P* = 0.275), and a *Z* statistic of 0.27.

### 6.2. Complementary Analysis

#### 6.2.1. Neuromuscular Blockade Agents (NMBA)

Subsequent analysis performed in relation to the dose of NMBA (rocuronium) administered showed that there were no significant differences between the two groups (>0.03 mg/kg and ≤0.03 mg/kg) at each of the four measurement times (*B*_1_, *P* = 0.056; *X*_1_, *P* = 0.290; *B*_2_, *P* = 0.441; *X*_2_, *P* = 0.791).

#### 6.2.2. Sedation Level

Statistically significant differences were noted in BIS during both noxious stimuli between participants with a baseline BIS ≥ 40 and those with a baseline BIS < 40. The median BIS increase was 16% superior during *X*_1_ and 18% superior during *X*_2_ in the group with a BIS ≥ 40 (Table 5).

## 7. Discussion

The aim of this study was to examine the relationship between the BIS and experimental noxious stimuli in adults under general anesthesia. BIS was found to significantly increase during both moderate and severe noxious stimuli (*X*_1_  and  *X*_2_) when compared with baseline BIS (*B*_1_  and  *B*_2_). However, at the best cut-off score, the BIS was not found to be very sensitive in distinguishing moderate from severe noxious stimuli. Although, it seems to be relatively specific.

In the present study, the BIS increased during both noxious stimuli. Previous studies in the operating room have also reported significant increases in BIS during noxious stimuli [22, 26]. Ekman et al. (2004) reported a significant increase in BIS during laryngoscopy (average of 6.3 (±6.6)) [22]. Similarly, Takamatsu et al. (2006) noted that there was a statistically significant increase in BIS in comparison with the absence of stimulation at a MAC of 1.3 [26]. Furthermore, these authors reported that at similar concentrations of sevoflurane, BIS increased when the intensity of an electrical stimulation increased (baseline (BIS = 36); 20 mA (BIS = 47); 40 mA (BIS = 50); 60 mA (BIS = 53); 80 mA (BIS = 57) [26]). In light of these results, the median increase in BIS during both noxious stimuli appears to be slightly lower in our study when compared to the Ekman et al. and Takamatsu et al. studies.

It is also known that the administration of NMBA such as rocuronium will diminish the expected increase in BIS in response to noxious stimuli [40]. In our study, participants received two possible doses of NMDA; however, no difference was found regarding the elevation of BIS between the two groups during both noxious stimuli. Therefore, we can conclude that the administration of NMBA was not a confounding factor in the interpretation of the findings of this study. However, the administration of NMBA may have attenuated BIS elevation amongst all participants.

Further analysis showed that there was a significant difference in BIS during both noxious stimuli in relation to whether the baseline BIS (*B*_1_  or  *B*_2_) was ≥40 (state of general anesthesia) or <40 (deep hypnotic state) with BIS increases statistically superior during both noxious stimuli in the group with BIS ≥ 40. Further analysis revealed that 60% of stable BIS in the current study were below 40, representative of a deep hypnotic state [34]. Considering that higher doses of sedative agents are correlated with lower BIS values [37], we may assume that the targeted stable BIS score in our protocol of 20–50, allowing for BIS < 40 in order to accommodate for local sedation practices, could have attenuated the increase of BIS during both noxious stimuli. In previous studies conducted in the operating room, targeted stable BIS values were higher, that is, between 40 and 60 [22, 25, 41]. With a higher targeted stable BIS score, we could possibly have expected a greater increase in BIS in response to noxious stimuli, such as what has been demonstrated in previous studies involving BIS in the ICU setting [18, 42].

To the best of our knowledge, this is the first study with a specific objective to examine the ability of the BIS to distinguish noxious stimuli of moderate and severe intensities by comparing the BIS with the gold standard in pain assessment, the patient's self-report. The AUC indicates that the BIS is not a valid instrument to distinguish different pain intensities in the context of the operating room at deep sedation levels.

This study was not without limitations. Several participants did not receive medication in accordance with the suggested anesthesia protocol which allowed a certain variation to accommodate local practice. However, all medications received were documented, and doses were calculated in relation to the participant's weight to identify the impact of such possible differences. Furthermore, average dose of fentanyl and of propofol, two medications known to possibly influence BIS, reflected suggested doses identified in the research protocol [37, 38]. In addition, considering the possible influence of NMBA, received by all participants, further analysis was performed to eliminate any potential influence in accordance to dosage.

Despite these limitations, this study allowed the observation of a relationship between BIS values and experimental noxious stimuli (determined by the gold standard of each subject's self-report) in adults under general anesthesia. Considering that objective of this study was to determine a relationship between two variables, it was of utmost importance to eliminate as many sources of confounding variables as possible such as those related to the heterogeneity and critically ill status of potential subjects. Therefore, a relatively homogeneous, healthy, population with an ASA I was chosen to allow control of these potential sources of confusion. Furthermore, several other potential confounding factors were controlled, such as the encouragement of a standardized regimen for medication administration. Moreover, the exclusion of patients taking opioids in the preoperative period, patients with diseases with systematic complications, and documentation of many other variables exposing their possible influence on the results of this study increases the internal validity of the study. It is important to mention that other variables which could possibly influence BIS values (electrical interference of OR equipment, etc.) that were not controlled in this study, arguably, should not have influenced the results of this study considering that any other potential confounding variable was present during all measurements for all participants.

## 8. Conclusion

In conclusion, moderate and severe experimental noxious stimuli led to significant increases in BIS values. However, the amplitude of this variation is probably too weak to be clinically useful in adjusting levels of analgesia in the context of the OR where patients are deeply sedated. Furthermore, the Se (40.0%) and Sp (73.3%) of the BIS in distinguishing moderate and severe intensities of experimental noxious stimuli are too weak to justify utilizing this score as a valid measure of pain in the OR. However, analysis on participants with a baseline BIS greater than 40 revealed higher increases in BIS values, suggesting that the increase in BIS values in response to a noxious procedure would quite possibly be higher in ICU patients with lighter sedation levels. Therefore, findings from this study support further investigation pertaining to the sensitivity and specificity of BIS in pain assessment in critically ill patients with lighter sedation levels. Such investigation would clarify the potential utility of the BIS in pain assessment in critically ill patients in the ICU.

## Figures and Tables

**Table 1:** Medication received by participants and MAC.

Values are mean (SD)	
Fentanyl	Suggested dose in protocol: 2-3 mcg/kg
	Dose received by participants: 2.3 mcg/kg (0.10)
Rocuronium	2 possible doses in protocol
	(i) 0.6 mg/kg: *n* = 20
	(ii) 0.03 mg/kg: *n* = 10
MAC	Ideal MAC: <1.3
	Average: 0.95 (0.19)

Description of medication administered to participants and MAC value before start of experimental phase. MAC indicates “minimum alveolar concentration of anesthetic.” Mcg indicated micrograms; kg is kilograms, *n* is the number of subjects having received the dose indicated.

**Table 2:** Relationship between BIS and experimental noxious stimui.

	Median	IQR	Wilcoxon signed-rank test
Moderate experimental noxious stimuli (*X*_1_)			
*B*_1_	35.00	25.75–42.56	*P* = 0.003
*X*_1_	40.00	28.50–46.00	

Severe experimental noxious stimuli (*X*_2_)			
*B*_2_	37.67	26.75–43.54	*P* = 0.007
*X*_2_	40.00	28.00–48.25	

Changes in BIS in response to moderate (*X*_1_) and severe (*X*_2_) experimental noxious stimuli. Median and interquartile ranges are indicated for the 4 observation periods (*B*_1_, *B*_2_, *X*_1_, and *X*_2_). For the statistical difference between the two groups of participants, the Wilcoxon signed-rank test was used with *P* values.

**Table 3:** Intersubject Delta BIS for both noxious stimuli.

	Median Delta Δ	IQR	Mann-Whitney *U* Test
*X*_1_ − *B*_1_ (Δ1)	1.7500	−0.1875 to +6.3125	*P* = 0.847
*X*_2_ − *B*_2_ (Δ2)	1.6650	−0.4350 to +7.4150	

Changes of BIS during each noxious stimuli. The median difference between BIS during moderate noxious stimuli (*X*_1_) and baseline 1 (*B*_1_) BIS and the difference between severe noxious stimuli (*X*_2_) and baseline 2 BIS (*B*_2_) were calculated as well as the IQR. The statistical difference between *X*_1_ and *B*_1_ (Δ1) and *X*_2_ and *B*_2_ (Δ2) was calculated with the Mann-Whitney *U* Test.

*X*_1_: moderate noxious stimuli (40/100); *B*_1_: baseline 1; Δ1: Delta 1; *X*_2_: severe noxious stimuli (70/100); *B*_2_: baseline 2; Δ2: Delta 2; IQR: Interquartile range.

**Table 4:** Sensitivity and specificity at different BIS values.

Criterion	Sensitivity	95% CI	Specificity	95% CI
≥16	100.00	88.4–100.0	0.00	0.0–11.6
>16	96.67	82.8–99.9	0.00	0.0–11.6
>19	96.67	82.8–99.9	3.33	0.08–17.2
>24	86.67	69.3–96.2	3.33	0.08–17.2
>25	83.33	65.3–94.4	10.00	2.1–26.5
>26	80.00	61.4–92.3	20.00	7.7–38.6
>27	80.00	61.4–92.3	23.33	9.9–42.3
>28	73.33	54.1–87.7	23.33	9.9–42.3
>29	70.00	50.6–85.3	26.67	12.3–45.9
>31	70.00	50.6–85.3	33.33	17.3–52.8
>34	56.67	37.4–74.5	33.33	17.3–52.8
>36	56.67	37.4–74.5	36.67	19.9–56.1
>38	50.00	31.3–68.7	40.00	22.7–59.4
>41	50.00	31.3–68.7	56.67	37.4–74.5
>42	43.33	25.5–62.6	63.33	43.9–80.1
>43	40.00	22.7–59.4	66.67	47.2–82.7
>45∗	40.00	22.7–59.4	73.33	54.1–87.7
>46	26.67	12.3–45.9	80.00	61.4–92.3
>47	26.67	12.3–45.9	86.67	69.3–96.2
>48	23.33	9.9–42.3	90.00	73.5–97.9
>49	16.67	5.6–34.7	90.00	73.5–97.9
>50	16.67	5.6–34.7	93.33	77.9–99.2
>51	13.33	3.8–30.7	93.33	77.9–99.2
>53	13.33	3.8–30.7	96.67	82.8–99.9
>54	6.67	0.8–22.1	100.00	88.4–100.0
>58	0.00	0.0–11.6	100.00	88.4–100.0

Sensitivity and specificity of BIS in distinguishing moderate from severe noxious stimuli at different thresholds of BIS (criterion) with 95% confidence intervals.

>: superior; <: inferior; CI: confidence interval.

∗Best cut-off ROC score. Threshold associated with maximization of the sums of sensitivity and specificity.

**Table 5:** Changes in BIS in relation to baseline BIS value (≥40 OU <40).

	Median	IQR	Mann-Whitney *U* Test
BIS during moderate noxious stimuli (*X*_1_)			
*B*_1_ < 40 (*n* = 18)	30.50	26.00–40.00	*P* = 0.00
*B*_2_ ≥ 40 (*n* = 12)	46.50	43.25–49.50	

BIS during severe noxious stimuli (*X*_2_)			
*B*_1_ < 40 (*n* = 18)	30.50	24.75–39.00	*P* = 0.00
*B*_2_ ≥ 40 (*n* = 12)	48.50	46.00–53.25	

Changes in BIS during moderate and severe noxious stimuli according to whether baseline BIS was ≥40 or <40 were determined. Median and IQR of BIS during both noxious stimuli are noted for each of the two groups. Statistical difference (*P* value) between both groups was then calculated for each of the noxious stimuli with the Mann-Whitney *U* Test.

*B*_1_: baseline 1; *B*_2_: baseline 2; >: superior; <: inferior; IQR: interquartile range; *n*: number of participants in each group.
